# Competent but complex communication: The phenomena of pheromone-responsive plasmids

**DOI:** 10.1371/journal.ppat.1008310

**Published:** 2020-04-02

**Authors:** Amy J. Sterling, William J. Snelling, Patrick J. Naughton, Nigel G. Ternan, James S. G. Dooley

**Affiliations:** Nutrition Innovation Centre for Food and Health (NICHE), Ulster University, Coleraine, Londonderry, Northern Ireland; Geisel School of Medicine at Dartmouth, UNITED STATES

## Abstract

Enterococci are robust gram-positive bacteria that are found in a variety of surroundings and that cause a significant number of healthcare-associated infections. The genus possesses a high-efficiency pheromone-responsive plasmid (PRP) transfer system for genetic exchange that allows antimicrobial-resistance determinants to spread within bacterial populations. The pCF10 plasmid system is the best characterised, and although other PRP systems are structurally similar, they lack exact functional homologues of pCF10-encoded genes. In this review, we provide an overview of the enterococcal PRP systems, incorporating functional details for the less-well-defined systems. We catalogue the virulence-associated elements of the PRPs that have been identified to date, and we argue that this reinforces the requirement for elucidation of the less studied systems.

## Introduction

The genus *Enterococcus* encompasses approximately 50 species renowned for their hardy nature and are found in a wide array of environments, from the human intestinal tract to soils in tropical and subtropical climates. Genome sizes range from 2.3 to 4.5 Mb and average 38% GC content [1,2]. Although originally regarded as commensal gut microbes, the enterococci have, over the past few decades, become recognised as major causes of healthcare-associated infections. Enterococcal infections are increasingly challenging to treat because of intrinsic antibiotic resistance possessed by *Enterococcus* spp. They can exhibit resistance to common antibiotics (ampicillin or penicillin) and easily acquire new antimicrobial resistances (AMRs) (e.g., to linezolid) [3–5]. Indeed, it has been noted that antimicrobial-resistant strains possess larger genomes than nonpathogenic enterococcal isolates [6]. Enterococci utilise mobile genetic elements (MGEs) such as transposons and plasmids to disseminate or acquire further resistance determinants and/or novel virulence factors [7,8]. Worryingly, some enterococcal plasmids are adapted to persist in a broad range of bacterial hosts, conveying traits across the boundary of a single genus. For example, a broad host range plasmid belonging to incompatibility group 18 was demonstrated to transfer vancomycin resistance (*VanA*) from enterococci to methicillin-resistant *Staphylococcus aureus* (MRSA) [9].

For certain plasmids that transfer only within the enterococci, a peptide pheromone signal is used to stimulate their distribution. This peptide pheromone is produced in, and released by, plasmid-free (recipient) cells and generates a plasmid transfer response in plasmid-containing (donor) cells [10–12]. The first pheromone-responsive plasmid (PRP) encountered was pAD1, but investigations of plasmid transfer have focused on one other plasmid—pCF10, which, unsurprisingly, remains the best characterised [13]. Less emphasis has been placed on the mechanisms underlying transfer within other systems, however, and in this review, we bring up to date what is known about PRP systems, placing their mechanistic details in the context of the well-understood pCF10 system.

### Significance of the PRP system

The PRP transfer system is highly efficient with conjugation reactions exhibiting an efficiency of up to 10^−1^ (one transconjugant per 10 donor cells) under ideal conditions in liquid matings [14,15]. Although our understanding of PRP transfer in the intestinal tract is not complete, data from mouse studies reveal that the transfer of pCF10 within the upper intestinal tract is high even in the presence of a competing microflora. The authors noted that the number of pCF10-containing cells increased during the experiment, which is in line with work by Licht and colleagues [16], who noted that transconjugants containing pCF10 persisted for longer than donor cells within the intestines of mini pigs. Similarly, transfer of pAD1 occurs at high rates within Syrian hamsters. In all cases, animal models were inoculated with donor and recipient strains each at 10^7^ colony-forming units (CFU) and above. Typical enterococcal numbers within humans range between 10^5^ and 10^7^ CFU per gram; thus, the abovementioned results reflect the transfer of PRP under conditions of dysbiosis [17–21].

### Transferrable phenotypes encoded by PRP

Numerous PRPs have been identified within enterococci to date (Table 1), and with more genome sequences available, the number of identified PRPs is likely to increase. Many PRPs directly transfer an assortment of growth promoters and virulence traits (e.g., bacteriocins and biofilm enhancers) into recipient enterococcal cells and may also contribute other advantages. For example, Hirt and colleagues observed an increase in transconjugants even under nonselective conditions in mice, leading them to hypothesise that plasmid transfer also conferred some unknown metabolic advantage to recipient cells [18,22,23]. The acquisition of several PRPs would theoretically benefit cells in their progression from commensal to pathogen by contributing factors that could promote survival in the host. To date, 35 PRPs have been identified: the majority of these are present in *Enterococcus faecalis*, with only a few described in *Enterococcus faecium*.

**Table 1:** Enterococcal pheromone-responsive plasmids.

Plasmid	Size (Kb)	Original Host	Pheromone (Sequence If Known)	Plasmid Features	Reference
pCF10	65	*E*. *faecalis* SF-7	cCF10 (LVTLVFV)	*Tn925* *uvrA*	[24,25]
pHKK703	55	*E*. *faecium* R7	cCF10	Mobilisation of pHKK702	[26]
pBRG1	50	*E*. *faecium* LS10	cCF10–like	*Tn1546*	[27]
pMG2200	106.5	*E*. *faecalis* NKH15	cCF10	*Tn1549* *bac41* UV resistance	[22]
pMB1	90	*E*. *faecalis* S-48	cCF10	BC-48	[28]
pAMS1	130	*E*. *faecalis* MC4	cCF10	*bacA- bacB* Chloramphenicol, streptomycin, tetracycline resistance	[29]
pPD1	58	*E*. *faecalis* 39–5	cPD1 (FLVMFLSG)	*bac21*	[30–33]
pMB2	58	*E*. *faecalis* S-48	cPD1	AS–48	[28,34]
pYI14	61	*E*. *faecalis* YI714	cPD1	*bac41*	[35]
pEJ97-1	11.3	*E*. *faecalis* EJ97	cPD1	Enterocin EJ97	[36]
pAD1	60	*E*. *faecalis* DS16	cAD1 (LFSLVLAG)	*uvrA* Cytolysin	[37,38]
pTEF1	66.3	*E*. *faecalis* V583	cAD1	*Tn4001* *qacZ* Erythromycin resistance	[6,39,40]
pMG2201	65.7	*E*. *faecalis* NKH15	cAD1	*ermB* Cytolysin	[22]
pBEM10	70	*E*. *faecalis* HH22	cAD1	*Tn4001* *bla*	[41]
pAMγ1	60	*E*. *faecalis* DS5	cAD1	*uvr* *hly–bac*	[31]
pJH2	59	*E*. *faecalis* JH1	cAD1	*hly–bac*	[42–45]
pIP964	65	*E*. *faecalis*	cAD1	*hly–bac*	[44]
pTW9	85	*E*. *faecalis*	cAD1	Tn1546 *hly–bac* *ermB*	GenBank: AB563188
pOB1	64.7	*E*. *faecalis* OG1	cOB1 (VAVLVLGA)	*hly–bac*	[22,46]
pYI1	58	*E*. *faecalis*	cOB1	*hly–bac*	[46]
pTEF2	57.7	*E*. *faecalis* V583	cOB1	Unknown	[39]
pAM373	36.7	*E*. *faecalis* RC73	cAM373 (AIFILAS)	*uvrA*	[47,48]
pAM368	107	*E*. *faecalis* 368	cAM373	*Tn1546-* like	[49]
pSL1	128	*E*. *faecalis* KV1	cSL1	*Tn1546-* like *ermB* *aph(3′)* *ant(6′)* *aac(6′)–aph(2′)*	[50]
pSL2	128	*E*. *faecalis* KV2	cSL1	*Tn1546-* like *ermB* *aph(3′)* *ant(6′)* *aac(6′)–aph(2′)*	[50]
pAM323	66	*E*. *faecalis* HH2	cAM323	Erythromycin resistance	[41]
pAM324	53	*E*. *faecalis* HH2	cAM324	None	[41]
pHKK100	55	*E*. *faecium* 228	cHKK100	*Tn1546*	[51]
pYI2	56	*E*. *faecalis*	cYI2	*hly–bac*	[46]
pYI17	57.5	*E*. *faecalis* YI717	cYI17	*bac31*	[52]
pAMγ2	Approximately 60	*E*. *faecalis* DS5	cAMγ2	None	[31,45]
pAMγ3	Approximately 60	*E*. *faecalis* DS5	cAMγ3	None	[31,45]
pBEE99	80.6	*E*. *faecalis* E99	Unknown	*uvrA* *bee* *bac41*–*like*	[23]
pLG1*	268	*E*. *faecium* UW2774	Unknown	*vanA* *ermB* Teicoplanin resistance *hyl*_*efm*_ Varied carbohydrate metabolism Heavy metal resistance	[53]
pLG2*	62	*E*. *faecium* UW3114	Unknown	*ermB* Tetracycline resistance	[54]

*Plasmid not formally proven as pheromone-responsive but that contains DNA-binding proteins, a T4SS, and other DNA transfer machinery homologous to that of the canonical pheromone-response plasmids.

Abbreviations: *aac(6′)–aph(2′*), broad substrate range aminoglycoside acetylase and phosphorylase; *ant(6′*), aminoglycoside nucleotidyltransferase; *aph(3′)*, aminoglycoside-modifying enzyme; bee, biofilm enhancer; *bla*, β lactamase; *ermB*/*C*, erythromycin resistance; *hly–bac*, haemolysin bacteriocin; hyl_Efm_, hyaluronidase; T4SS, type 4 secretion system; Tn, transposon; *uvrA/*B, UV resistance; *vanA/B*, vancomycin resistance

#### Antibiotic resistance

Several of the PRPs identified to date contain transposable elements (Table 1) that confer antibiotic resistance. For example Tn1546, encoding *vanA*, is found in a few PRPs, although so far only one example of a *vanB*–encoding transposon (Tn1549) has been identified in enterococcal PRPs [22,27,49–51]. Either *van* gene confers vancomycin resistance, and other transposable elements confer resistance to other antibiotics, including gentamicin and kanamycin (Tn4001) as well as erythromycin and tetracycline (Tn925) [24,55]. Certain PRPs possess toxin/antitoxin (TA) systems that ensure plasmid inheritance within a population even in the absence of antibiotics. The best studied of these is the type I TA *par* system in pAD1 encoding the faecalis plasmid-stabilizing toxin (Fst); however, this system has been reviewed elsewhere [56]. Fst-like peptides can be found within a number of gram-positive chromosomes (such as EF0409 of *E*. *faecalis* V583) as well as several PRPs [57]. Both pAMS1 and pTEF2 contain Fst-like peptides ensuring their continuation within a cell population [56]. Similarly, pTW9 encodes the type II epsilon/zeta TA loci—a widespread TA system that maintains plasmids in both gram negatives and gram positives [58].

Within PRPs, there is clearly a reservoir of antimicrobial-resistance genes and potential for their transfer and maintenance within a population. However, PRP transfer outside of enterococci into unrelated bacteria will likely be impeded by several hurdles. For example, difficulties in maintaining cell-cell contact could result in ineffective mating, and furthermore, new hosts may contain CRISPR-Cas systems that destroy ‘foreign’ nucleic acids. Additionally, a number of host-specific factors, such as the failure of plasmid initiator proteins to interact successfully with host proteins, may also limit plasmid replication [59].

Coupled to their extensive genome-encoded intrinsic AMR, enterococcal acquisition of PRPs and their associated antibiotic resistance genes could have environmental, agricultural, and medical implications [3,4]. Resistant strains from environmental and agricultural sources could act as direct causes of infection or as vectors for transmission of resistance genes into human pathogens. In healthcare, for example, infections caused by vancomycin-resistant enterococci (VRE) are associated with greater mortality rates than those caused by non-VRE [60,61]. If enterococci can acquire additional resistance genes through a diversity of PRPs, and stably maintain them, current treatment approaches that rely on the synergistic effect of multiple antimicrobials for VRE (β-lactams combined with aminoglycosides, for example) may become futile.

#### Bacteriocins

Bacteriocins are ribosomally produced heat-stable proteins or peptides that allow competitive inhibition of other bacteria through disruption of the cell envelope. Enterococcal genomes encode bacteriocins of varying classes, providing them with a major advantage in highly competitive environments such as the human intestine [62,63]. Two PRPs, pAD1 and pMG2201, both encode the class I bacteriocin cytolysin (Table 1) that is associated with a poorer clinical outcome in cases of septicaemia [64]. Furthermore, it has been demonstrated that in a mouse intestine model, *E*. *faecalis* harbouring the bacteriocin 21–encoding PRP, pPD1, outcompetes indigenous *E*. *faecalis* cells that lack the plasmid [65].

#### UV resistance

Some PRPs, such as pAD1, encode UV resistance via *uvrA*, which shows homology to the UmuC protein of *Escherichia coli* [66]. UV damage results in an increased mutational rate as a result of error-prone repair, and Miehl and colleagues have shown that UV-irradiated, UV-resistant *E*. *faecalis* was more likely to develop spontaneous mutations facilitating resistance to antibiotics that they were subsequently challenged with, compared to non-UV-resistant cells [67].

### Inter- and intraspecies dissemination of PRP

PRP transfer between closely related enterococcal species is unsurprising, and many cases of transfer between *E*. *faecalis* and *E*. *faecium* have been reported [27,68]. However, in liquid matings, higher rates of PRP transfer from *E*. *faecalis* to *E*. *faecium* are observed rather than vice versa [69,70]. This interspecies transfer of PRP is undoubtedly of concern as many environments host multiple bacterial species simultaneously. Enterococci can also share DNA with closely related bacteria including members of the staphylococci, in which the transfer of *vanA*-mediated vancomycin resistance, albeit in pheromone-independent matings, has been reported [71,72]. In addition, staphylococci could conceivably take up enterococcal PRPs, and thus the possibility of PRP-mediated transfer of vancomycin resistance to MRSA is not completely irrational [49,59]. *S*. *aureus* can produce a cAM373-like peptide, and under laboratory conditions, *E*. *faecalis* containing pAM368 (Tn1546, encoding *vanA*) can respond to *S*. *aureus–*generated cAM373. Intergeneric studies have utilised pAM373 derivatives and coresident plasmids because although streptococcal uptake of PRPs may occur, the pAM373 replicon is nonfunctional in *S*. *aureus* [73]. The lipoprotein precursor from which the enterococcal and staphylococcal cAM373- and cAM373-like peptides, respectively, are derived share no sequence homology. It has, accordingly, been postulated that the similarities between these mature heptapeptides are simply coincidental [73,74].

PRPs can also influence the transfer of other mobile DNA elements and plasmids. For instance, the 153-kb *E*. *faecalis* pathogenicity island (PAI) contains numerous other virulence-enhancing factors and is capable of intra- and interspecies transfer, aided by the plasmid pTEF2 [54,59]. In *E*. *faecium*, pHKK703 can mobilise nonconjugative plasmid DNA (pHKK702), further widening the collection of potentially pathogenic traits available for transfer to recipient cells [26]. Plasmid pTEF2 can also mobilise a substantial portion of the V583 chromosomal DNA (up to 857 kbp) to a recipient *E*. *faecalis* chromosome. Here, the authors postulate that chromosome-to-chromosome transfer accounts for the emergence of virulent hospital-derived *E*. *faecalis* strains and this is facilitated at least in part by PRPs [75]. Interspecies transfer of chromosomal DNA is rare, as sequence homology is required for the insertion of new DNA into the chromosome—a barrier for even closely related species. Thus, the contribution of PRP to interspecies DNA mobilisation will be limited, but the role of PRP in DNA transfer within *E*. *faecalis* is less constrained.

## Pheromone-induced conjugal transfer of plasmid DNA

PRP transfer occurs between donors (plasmid-containing) and recipient (plasmid-free) cells via the actions of the plasmid-encoded machinery. During liquid mating, contact between the donor and recipient cells is maintained by the aggregation substance (PrgB) after cells collide (see section on Adherence). PrgB is required for successful plasmid transfer in liquid; however, in solid mating, the solid surface supports cell-to-cell contact without the same requirement for the aggregation substance [76]. Transfer is initiated by high concentrations of recipient cell–produced peptide pheromone, which generally correlates with a rise in recipient cell density. The inducing pheromones are transcribed from both recipient and donor cell chromosomes for pCF10 [31]. However, donor cells produce corresponding inhibitor peptides that competitively inhibit the inducing pheromones [25].

Unlike pCF10, production of inducing pheromones cAD1 and cPD1 in pAD1 and pPD1 is shut down in donor cells [32,77]. The following section will consider the key interactions between plasmid-encoded components that are necessary for the pheromone response and encoded by the pheromone-responsive genes (*prg*).

### The rival peptides

Plasmid transfer depends on the intracellular concentration of both the pheromone and the inhibitor peptides for each plasmid. For example, pCF10 transfer is induced by cCF10 and is inhibited by the plasmid-encoded iCF10 peptide. These competing peptides share various features such as their size (between 7 and 8 amino acids in length), composition, and processing, but contrast in their cellular role. The majority of enterococcal PRPs respond to one of five identified pheromones (Table 2).

**Table 2:** Enterococcal pheromone and inhibitor peptides identified to date.

Plasmid	Pheromone	Inhibitor	Reference
pCF10	…LLMAGLVTLVFVLSA**C**GT…	…AVVIAITLIFI	[24]
pAD1	…FAAIALFSLVLAG**C**G…	…PLITLFVVTLVG	[78]
pPD1	…GSGLLFLVMFLSG**C**VKTG…	…ALLFALILTLVS	[79]
pOB1	…VITVAVAVLVLGA**C**GNKK…	…SLTLILSA	[80]
pAM373	…FSLLGAIFILAS**C**GIGK…	…SIFTLVA	[47]

Mature forms of pheromone and inhibitor peptides are underlined, with conserved cysteine residues highlighted in bold.

**Fig 1:**
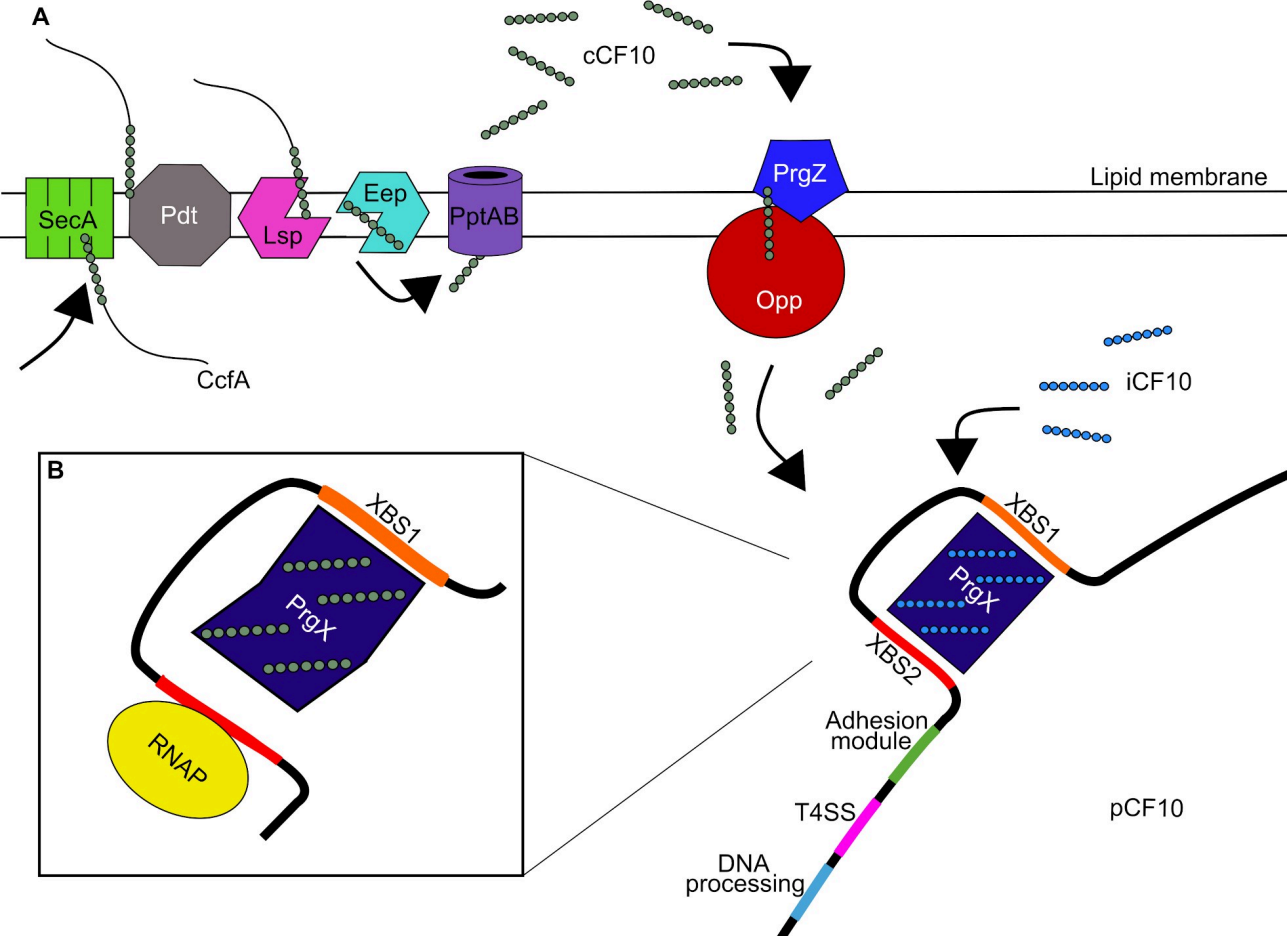
Model for induction of conjugation genes in pCF10. (A) Chromosomally encoded CcfA lipoprotein (grey) is exported to the extracellular region, where it is attached to the lipid membrane by Pdt. Subsequently, Lsp II cleaves the precursor on its cysteine residue before mature cCF10 is cut from the lipoprotein by Eep. Hydrophobic cCF10 is actively transported out of the cell through PptAB. Exogenous cCF10 (grey) is recognised by pCF10-encoded and externally presented PrgZ and is then passed to Opp for active uptake. In cCF10 absence, the PrgX tetramer is bound in a 1:1 ratio with iCF10 peptides (blue) and maintains tight binding to the pCF10 DNA through binding sites XBS1 and XBS2, thereby sterically inhibiting the binding of RNAP. (B) Within the induced state, the PrgX/cCF10 complex replaces the PrgX/iCF10 complex on the pCF10 DNA. The PrgX/cCF10 complex fails to maintain tight binding to XBS2, allowing RNA polymerase to access and then transcribe the downstream conjugation inducing genes. Eep, enhanced expression of pheromone; Opp, oligopeptide permease; Pdt, prolipoprotein diacylglyceryl transferase; Prg, pheromone-responsive gene; RNAP, RNA polymerase; T4SS, type 4 secretion system; XBS, PrgX binding site.

To produce mature cCF10, the pheromone must be cleaved from a lipoprotein precursor in which it exists as the last seven residues of the lipoprotein signal peptide. The function of these lipoproteins has not been fully determined, although their presence (bar the pheromone) is nonessential for PRP uptake. CcfA, the precursor to cCF10, exhibits similarity to the gram-negative translocase YidC [10] and is secreted in a SecA-dependent manner prior to being anchored to the membrane by a prolipoprotein diacylglyceryl transferase (encoded by *ef1748*) cleavage reaction [81,82]. These anchored inducing peptides are then cleaved once more by the lipoprotein signal peptidase II (encoded by *ef1723*) at conserved cysteine residues (Table 2) [83]. Both inducer and inhibitor precursors are then recognised by the membrane-localised metalloproteinase, enhanced expression of pheromone (Eep, encoded by *ef2380*), via their N termini [84,85] (Fig 1).

Processing and release of the active moieties by Eep is a shared step in the production of both inducers and inhibitors for pCF10, pAD1, pPD1, and pOB1, but not for pAM373 [11]. Mature—and very hydrophobic—pheromone peptides are then actively transported out of the cell via the ATP-binding cassette (ABC) transporter PptAB, which is known to transport cCF10-, cOB1-, and cAM373-inducer peptides [11,80,85,86].

### Peptide detection and import

Detection of extracellular pheromones and their corresponding inhibitors is mediated by plasmid-encoded receptors but also requires a chromosomally encoded system for active peptide uptake.

### The extracellular pheromone-binding protein

Both inducer and inhibitor peptides compete for binding sites on the same extracellular receptor. The plasmid-encoded cCF10 receptor, PrgZ, exhibits a slightly higher affinity for inducer cCF10 over inhibitor iCF10. The binding pocket within PrgZ is narrow and both cCF10 and iCF10 possess a conserved threonine at position three. This very hydrophilic residue is hypothesised to be important for PrgZ-specific interactions, with peptide specificity thought to arise from the size, shape, and charge of amino acid side groups [12,87].

Plasmids pAD1 and pPD1 both encode a protein designated TraC as the surface receptor, and these share 71% sequence identity [88]. The pAD1- and pPD1-encoded TraC also share 72% and 87% identity, respectively, to PrgZ. Upon recognition of the peptides, PrgZ/TraC then recruit the chromosomally encoded oligopeptide permease (Opp) machinery for active transport of peptides into the cell [12,88,89].

### The Opp import system

The Opp system belongs to the ABC family of transporters and comprises five proteins encoded by a single operon [90]. The lipoprotein OppA is usually responsible for capture of target peptides; however, in the pCF10 pheromone system, PrgZ dominates in this role. OppA is not selective with respect to substrate binding and is able to bind a range of peptides up to 35 residues in length [91,92], and therefore, the selectivity of the PRP system derives from the main peptide receptor PrgZ/TraC. Subsequently, PrgZ/TraC deliver bound peptides to the inner membrane for translocation by OppB and OppC proteins, with energy for the process coming from the cytoplasmic ATP-hydrolysing components OppD and OppF [93]. Interestingly, *E*. *faecalis* mutants lacking PrgZ/TraC were still inducible, albeit they required a 4-fold higher exogenous concentration of their respective pheromone than the wild type. Pheromone recognition in the absence of PrgZ/TraC is thought to occur through OppA alone [32,89].

### Induction of conjugative genes

The active uptake of pheromones by donors allows cells to respond to even low levels of surrounding pheromones: under laboratory conditions, this is equated to around five pheromone molecules per donor [94]. In the case of cCF10, this peptide pheromone prevents the transcriptional regulator PrgX from continuing to repress the conjugation genes, thereby allowing conjugation to occur.

Homologues of PrgX (*TraA* genes) are found within pPD1 and pAD1 plasmids, highlighting the importance of the repressor function in the uninduced state [77,95].

Recent work [96] suggests that donor cells within a population contain slightly different ratios of the inducing and inhibiting pheromones as well as differences in concentrations of the internal repressor PrgX. This leads to a stochastic cell distribution with some cells exhibiting quicker responses to cCF10 than others [96].

#### The uninduced state

PrgX is a dimeric DNA-binding protein encoded on pCF10 (*TraA* in pPD1 and pAD1) and belongs to a family of peptide-binding regulators (RRNPP) [97,98]. Two dimers of PrgX (tetramer) bind to PrgX binding site (XBS) operator sites XBS1 and XBS2 on pCF10. XBS1 and XBS2 are 70 bp apart and on the same DNA strand of pCF10, and thus, tetramer binding forms a loop in the DNA. XBS2 overlaps the *PrgQ* promoter (P_Q_); hence, both PrgX and RNA polymerase compete for binding [99–102]. Until recently, it was not known how two very similar pheromone peptides could regulate the transcription of the pCF10 conjugative genes [103]. In the uninduced state, the PrgX tetramer configuration is promoted by the binding of iCF10 to PrgX in a 1:1 ratio (Fig 1) [101]. Binding of iCF10 to PrgX is stronger (than cCF10 binding) and stabilises the alignment of all PrgX monomers on the same spatial plane, thereby favouring a tight bond between PrgX and XBS2, which prevents RNA polymerase binding to P_Q_ through steric hindrance. Thus, iCF10 favours repression of the conjugation genes encoded in P_Q_ [104–106]. The PrgX/iCF10 complex can inhibit 90%–95% of P_Q_ transcription [102], and thus—although the precise nature of the PrgX interaction with DNA is yet to be fully elucidated—iCF10 functions to inhibit conjugative transfer of pCF10 when the donor cell population is higher than that of the recipient [107].

#### The induced state

In the induced state, PrgX/cCF10 replaces PrgX/iCF10 on the plasmid DNA. Within PrgX/cCF10, the PrgX tetramer proteins are no longer spatially aligned as they are in the iCF10 complex, with one dimer being rotated out of the alignment plane (Fig 1). The rotational stress on the DNA molecule renders PrgX unable to maintain tight binding with XBS2, enabling RNA polymerase to compete successfully for binding at P_Q_. This allows transcription of the conjugation-initiation genes *prgR-prgT* [104–106]. Chen and colleagues note, however, that further experiments are required to elucidate whether PrgX tetramer dissociation also contributes to transcription initiation [106].

Conversely, in pAD1-containing cells, conjugation initiation relies simply upon TraA dissociation from DNA upon cAD1 binding [77]. Distinct yet again from this is TraA in pPD1, in which three DNA-binding sites, rather than two, control conjugation gene regulation [95].

### Conjugation and plasmid transfer

The *PrgQ* operon, under control of P_Q_, contains all the genes responsible for pCF10 transfer in its 3′ end. These genes can be organised into four functional categories (Fig 1). The 5′ region is regulatory and is followed by the adherence module, which mediates cell aggregation. The distal end of the operon encodes the plasmid mobilisation genes, consisting of a type 4 secretion system (T4SS) utilised as a bacterial mating channel between donor and recipient cells, in addition to plasmid processing genes [20].

#### Adherence

In pCF10, the *prgB* gene encodes the aggregation substance PrgB, which becomes dispersed evenly over the cell surface with the exception of the septal ring [108]. This protein is homologous with Asp1 in pPD1, Asa1 in pAD1, and Asa373 in pAM373, and all contain an LPXTG motif that facilitates cell wall anchoring [95]. PrgB and Asp1 contain two arginine–glycine–aspartic acid (RGD) elements, one towards the N terminus and the other at the C terminus (Asa1 contains only one RGD; Asa373 does not contain an RGD) [48,109] (UniProt: P17953 and Q47766). These RGD motifs permit attachment to human integrin molecules; however, the N-terminal RGD in PrgB is more important for this than the C-terminal motif [110]. Endocarditis studies in rabbits and internalisation studies in human neutrophils have, however, emphasised that expression of PrgB by *E*. *faecalis* increases virulence, presumably by facilitating interactions with host cells via the RGD motifs [111]. *E*. *faecalis* generates the aggregation substance on its surface following exposure to both bovine and human serum—occurring in the absence of pheromone and for, presumably, the sole purpose of increasing virulence [112]. Domain Asc10_(156–358)_ within PrgB mediates binding to enterococcal cell walls, thus promoting cell-cell contact between donor and recipient cells, an observation confirmed by mutational studies that demonstrated a 100-fold reduction in pCF10 transfer in a *prgB* deletion mutant. Cell-to-cell adherence via PrgB is vital for successful plasmid transfer in liquid matings, but PrgB is not required for solid-surface matings [113].

#### Plasmid processing

pCF10 plasmid transfer is initiated by the accessory protein PcfF (belonging to the MobC accessory protein family) and the relaxase PcfG. TraX is the relaxase in pAD1 and is related to the relaxase from pAM373 (Orf8); however, both TraX and Orf8 share very little amino acid sequence homology with PcfG on pCF10 [114].

PcfF and PcfG function by binding to the origin of transfer (*oriT*) and cleave pCF10 within the nick region releasing the DNA strand to be transferred (T strand) [106,115]. Following cleavage, PcfG remains attached to and stabilises the 5′ end of the T strand, forming the PcfG–T strand intermediate. It is thought that the role of PcfG is to guide the T strand through the mating channel and into the recipient cell, and it is also suggested that PcfG stimulates recircularization of the transferred plasmid DNA, given the observation that it can rejoin *nic* sites in vitro [106].

Plasmid transfer has scarcely been characterised for other plasmids, though there are data available on pAD1, which has two oriT sites, the second of which is thought to be favoured for interspecies transfer [48,116]. Plasmid processing genes within several PRPs show limited homology, and indeed, the pCF10 relaxase PcfG is more closely related to the *Lactococcus lactis* pRS01-encoded relaxase LtrB, with which it shares 52% amino acid identity [117]. Analysis of sequenced PRPs indicates that regulatory regions within PRPs derive from a shared ancestor (within at least pCF10, pAD1, pAM373, and pPD1). However, plasmid evolution through incorporation of new DNA and frequent recombination has given rise to a diverse set of plasmids with vastly different processing modules [24]. This functional gain accounts for the number of antibiotic resistance genes and virulence factors encoded on many PRPs [22].

#### T4SS

T4SSs (reviewed extensively elsewhere [118]) are versatile and can function as conjugative systems, substrate uptake mechanisms, or general translocators. The pCF10 T4SS encoded between *prgF–pcfC* shows strong homology to the VirB/D4 T4SS found in the gram-negative *Agrobacterium tumefaciens* [24]. Openings in peptidoglycan are essential for T4SS assembly, and PrgK, which contains peptidoglycan hydrolase domains, is suggested to stabilise the junction between mating cells [119]. The hexameric PcfC (VirD4-like) and PrgJ (VirB4-like) ATPases are thought to provide energy for the physical transfer of pCF10. The structure of PcfC has been elucidated, revealing a 7-α helix component that confers substrate specificity, in addition to a channel-activation function [120]. The current working theory is that PcfC (a type IV coupling protein) recruits the PcfG–T strand intermediate via PcfG and delivers the complex to PrgJ. However, whether the PcfG–T strand intermediate is targeted via PrgJ to the recipient through the PcfC hexamer or whether this occurs through the T4SS inner membrane complex is still unknown [103,121,122].

### System maintenance and prevention of self-induction

The act of conjugation places a large metabolic burden on donor cells, and thus, tight regulation of PRP conjugative genes is necessary. This control is mediated at the level of transcription by independently transcribed short RNAs and also via negative regulation of *prgB* expression.

#### RNA-mediated control

Control of conjugation at the level of transcription is directly influenced by the PrgX/TraA molecular repressor in addition to levels of exogenous pheromone (Fig 1). In pCF10-containing cells, the uninduced state is characterised by significant intracellular quantities of short Q (Q_s_, 380-nucleotide [nt] RNA), from the P_Q_ regulatory region. On the 3′ end of the Q_S_, RNA is an inverted repeat sequence (IRS1) of a putative terminator [123]. To induce plasmid transfer, however, transcription must continue past IRS1 to the conjugation genes. Nascent PrgQ can form two secondary structures, one of which promotes transcriptional termination at IRS1, whereas the other encourages antitermination and thus permits transcription of conjugation genes. On the opposite DNA strand of pCF10 and 220 bp upstream is the promoter P_X_, from which P_X_ Anti-Q is transcribed, in addition to the mRNA for PrgX (or TraA for pAD1) from the 3′ end [123,124]. Anti-Q RNA (mD in pAD1) is unaffected by cCF10 presence, possesses a polyuridine tract, has a branched secondary structure, and is antisense to PrgQ mRNA. Anti-Q RNA therefore interacts with nascent PrgQ transcripts, favours the formation of the termination configuration, and prevents transcription past IRS1. Together, these prevent unnecessary transcription of conjugative genes from pCF10 [125].

In the presence of recipient cell–generated cCF10, however, the quantity of nascent PrgQ transcripts increases, but the level of Anti-Q RNA remains constant. The surplus PrgQ RNA takes the antitermination form that allows transcription to continue past IRS1 in order to produce the 530-nt long Q (Q_L_) RNA. Q_L_ is hypothesised to combine with ribosomes to effect the translation of mRNA for conjugation components [123–125].

pAD1, however, makes use of TraE1 (promoting conjugative gene transcription), which is repressed by TraA. Similarly, two transcriptional termination sites are present on pAD1 (T1 and T2) [125,126].

#### Aggregation substance regulation

Recently, Bhatty and colleagues [127] identified PrgU as a regulatory protein in pCF10. The *prgU* gene is located within the adherence module; however, it can also function as a negative regulator of the aggregation substance, PrgB. A lack of PrgU (and hence overproduction of PrgB) in the presence of cCF10 results in serious disruption of cell growth in deletion mutants [127]. How PrgU interacts with and regulates PrgB is yet to be elucidated, but structural analysis has shown that PrgU possesses a pseudouridine synthase as well as an archaeosine transglycosylase (PUA) fold, both known to be associated with RNA binding. Bhatty and colleagues suggested that, on this basis, PrgU may interfere with PrgB mRNA or alternatively work to stabilise the Q_s_ and Anti-Q interaction [127].

The importance of this regulation is underlined by the presence of *prgU* homologues within other PRPs. Plasmids encoding genes of similar amino acid sequence to *prgU* include pTEF2 (100% identity), pBEE99 (97% identity), pTEF1 (96% identity), pAD1 (96% identity), and pTW9 (96% identity).

#### Endogenous pheromone degradation

PrgY is a pCF10-encoded membrane-localised peptidase that is thought to function extracellularly to degrade endogenously produced donor cell cCF10. PrgY is homologous to TraB within pAD1 and pPD1 plasmids, although no PrgY homologue exists within pAM373 [32,48,85,128]. PrgY is similar the human Tiki metalloprotease, but the PrgY active site exhibits no homology to other cCF10 binding proteins (for example, PrgZ or PrgX). It has been suggested that PrgY binds to and degrades cCF10 after it has been actively transported out of donor cells, thus preventing increases in the concentration of cell wall–associated pheromone and ultimately reducing the probability of these peptides being recognised by pheromone receptors on other donor cells [129,130].

## Future directions

The presence of TA systems of various classes within several PRPs could facilitate foundation work on chromosomal TA system exploitation given the high level of homology between many chromosomal and plasmid TA systems. Some data appear to suggest VRE contain a greater assortment of TA systems over susceptible enterococcal isolates; thus, TA systems have been suggested as possible antimicrobial targets [131]. Kang and colleagues discussed the design of small molecules to interfere with the TA complex, allowing for activation of the toxin and leading to cell death. The authors also suggest an approach to starve cells of antitoxin by preventing transcription of the TA system through binding of a molecule to the system promoter [132]. Importantly, TA mechanisms are not homologous with any human systems, reinforcing their possible therapeutic exploitation.

PRPs have the potential to contribute to the rising levels of resistance observed in healthcare and environmental enterococcal strains. However, our knowledge of PRP transfer under conditions reflective of environments native to *Enterococcus spp*. is incomplete. Baquero and colleagues extensively reviewed the need for new drugs or strategies to limit the spread of AMR—and the authors suggest that in the case of PRPs, this could consist of conjugation inhibitors [133]. Indeed Kohler and colleagues recently noted that pheromone diversity between PRPs will limit broad-spectrum peptide mimicry inhibitors [99]. Thus, work aimed at more widely conserved regions (such as PrgU and its homologues) may provide the basis for a one-size-fits-all transfer inhibitor. However, development of such an inhibitor will require full investigation of the less well-studied PRPs considered within this review. Such work could contribute significantly to the generation of inhibitors and would be crucial for the control of PRP-mediated AMR and vital in prohibiting PRP distribution.
